# Extraction and Characterisation of African Star Apple (*Chrysophyllum albidum*) Seed Oil and the Adsorptive Properties of the Fruit Shell in Ghana

**DOI:** 10.1155/2019/4959586

**Published:** 2019-04-01

**Authors:** Michael A. Anang, Michael Oteng-Peprah, Kwasi Opoku-Boadu

**Affiliations:** ^1^Department of Chemistry, Industrial Chemistry Unit, School of Physical Sciences, University of Cape Coast, Cape Coast, Ghana; ^2^Department of Chemistry, Water and Sanitation Unit, School of Physical Sciences, University of Cape Coast, Cape Coast, Ghana

## Abstract

This research work was undertaken to determine the physicochemical parameters of oil from the seeds of African Star Apple (*Chrysophyllum albidum*) and further evaluate the adsorptive properties of the fruit shell. The oil was extracted using hexane with the soxhlet apparatus at a temperature of 65°C for 4 hours. The results showed an average oil yield obtained of 11.6%, specific gravity of 0.92kg/m^3^, the refractive index of 1.464 at 30°C, an acid value of 7.72 mg KOH/g, a free fatty acid value of 3.16 g/100g, saponification value of 200.56 mg KOH/g, and an iodine value of 70.64 g/100g. A Fourier Transform Infrared (FTIR) study on the oil identified some triglycerides, carbonyl, alkane, and alkene compounds. Adsorptive studies of the fruit shell for the removal of dye were also performed after chemical activation with CaCl_2_, MgCl_2_, and ZnCl_2_. The kinetics of the adsorption favoured a pseudo-first-order reaction pathway for CaCl_2_ with R^2^ of 0.941 while ZnCl_2_ and MgCl_2_ favoured a pseudo-second-order reaction pathway with R^2^ of 0.914 and 0.973, respectively.

## 1. Introduction

There are several fruits in Ghana that have essential oils which are of great medical importance. The African Star Apple (*Chrysophyllum albidum*) is a kind of fruit that is grown in Ghana. The tree grows as a wild plant and belongs to the family of Sapotaceae which has up to 800 species and constitutes almost half of the order [1]. It is a small-to-medium tree species, up to a height of 25-37 m having a mature girth varying from 1.5 to 2.0 m [2]. The fruits are not usually harvested from the trees, but left to drop naturally to the forest floor where they are picked [3]. Most plant seeds are a source of essential oils. Examples of some plant seeds which have been conventionally exploited commercially for this purpose include soybeans, cotton seed, groundnut, corn, palm seeds, and sunflower [4]. Oil from plant and animal are employed in the formulation of foods, cosmetics, and drugs in many industrial activities [5]. Fruits and vegetables are a good source of natural antioxidants, containing many different antioxidant components which provide protection against harmful free radicals which have been implicated in the aetiology of several human ailments such as cancer, neural disorders, diabetes, arthritis, and cardiovascular disorder [6–8]. An investigation on the antioxidant and food value of* Chrysophyllum albidum* showed the plant contains some phenol, flavonoid, anthocyanin, and proanthocyanidin and also a high antioxidant value [9]. Generally, the roots, barks, and leaves of* Chrysophyllum albidum* are widely used as an application to sprains, bruises, and wounds in southern Nigeria [10]. The seeds and roots extracts of* Chrysophyllum albidum* are used to arrest bleeding from fresh wounds and to inhibit microbial growth of known wound contaminants and also enhance the wound healing process [11]. The oil of* Chrysophyllum albidum* has been extracted from the corresponding seeds in a soxhlet extractor with hexane (boiling point range: 55°C-65°C) and analyzed for moisture content, pH, specific gravity, saponification value, refractive index, peroxide value, acid number, free fatty acid, and iodine value by [12]. Their results showed that the oil yield was of 21.57% and acid values were of 2.87. Similarly, [13] investigated into the extraction and characterisation of the seed oil. Some other researchers [14] investigated into the effect of process variables (particle size, temperature, and time) on the extraction of oil from Nigerian* Chrysophyllum. Albidum* to determine the optimum conditions for the extraction of the oil and also to characterize the oil extracted and determine its physicochemical properties. Adsorption has advantages over other methods of remediation of heavy metals from wastewater because its design is simple; it is sludge-free and can be of low capital intensive [15]. The most widely used adsorbent is activated carbon [16]. Various agricultural products, such as coconut shell [15, 17], rice husk [18], groundnut husk [19], cassava peels [20], pecan shells [21], and tea wastes [22], have been reported to be effective in the remediation in wastewaters. The seeds of* Chrysophyllum albidum* have been used in the adsorption of lead Pb from industrial wastewater by some researchers [23] where the effects of pH, contact time, and adsorbent mass were monitored. Steam-activated carbon prepared from* Chrysophyllum albidum* seed shell for the adsorption of Cadmium in wastewater (kinetics, equilibrium, and thermodynamic studies) was also investigated by [24] and was observed to be a potential sequester of Cadmium in wastewater. Studies were conducted by [25] on the effect of pH on the sorption of cadmium (ll), nickel (II), lead (II), and chromium (VI) from aqueous solutions by African white star apple (*Chrysophyllum albidum*) shell and realised the process is highly pH-dependent. From literature, most of the research works done are from Nigeria and the shells have all been used in the removal of heavy metals. The aim of this research paper is to extract and characterise oil from* Chrysophyllum albidum* fruit seed from Ghana and use the seed shell as an adsorbent in the removal of methyl orange (dye) from aqueous solutions.

## 2. Experimental Procedure

### 2.1. Fruit Collection and Seeds Preparation

Fresh ripped fruits of African Star Apple were bought from some local market sellers at the University of Cape Coast Science market which is located in the Southern part of Ghana. This is a seasonal fruit that is available during the dry seasons. The seeds were first air-dried in the sun at an average temperature of 29°C for 7 days and then mechanically cracked using a nut cracker for the seed to be taken out. The dried seeds were further air-dried for 5 days and then oven-dried at a temperature of 100°C for 24 hours in a laboratory oven (MMM Medcenter Ecocell 55).

### 2.2. Oil Extraction and Concentration Procedure

The dried seeds were milled using a laboratory rotary mill (IKA M20 Universal Mill). An amount of the milled seed was used for the Soxhlet extraction [26]. A round bottom flask containing analytical grade N-Hexane (99%) was fitted with a reflux condenser to the top. This was placed in a heating mantle at 65°C and the liquid condensate dripped into the thimble which contained the milled sample. The extract seeped through the pores of the thimble and filled a syphon tube and this was allowed to continue for 6 hours. The extract was then heated to recover the solvent with a rotary evaporator (R00102439, 50W/15A) leaving behind the extracted oil. The flask was then allowed to cool and the percentage yield was determined.

The refractive index, viscosity, saponification values, acid value, iodine value, free fatty acid value, specific gravity, and other parameters of the oil were determined using AOAC (2000) [27].

### 2.3. Infrared (FTIR) Analysis Coupled with Fourier Transform

The use of Fourier Transform Infrared (FTIR) spectrometer to determine the functional groups has been reported by various researchers [28, 29]. It was performed to determine the various functional groups of the chemical components using the Fourier Transform Spectroscopy Model I-R Prestige 21 Shimadzu.

### 2.4. Shell Preparation Prior to Adsorption Experiment

Seed shells of the* Chrysophyllum albidum* were air-dried for 14 days to constant weight and ground into powder. It was further air-dried for 7 days and then oven-dried in a laboratory oven (MMM Medcenter Ecocell 55) at 105°C for 8 hours to reduce the moisture content. It was then pyrolyzed in a furnace (Nabertherm, LE140K1BN, 230V, 1/N/PE) at a temperature of 500°C for two (2) hours. During pyrolysis, nitrogen gas at a flow rate 0.1m^3^/hr was used as purge gas. The pyrolyzed shells were milled into* a* powdery form and sieved with a laboratory mesh of size +500*μ*m.

The chemical activation of the pyrolised and powdered* Chrysophyllum albidum fruit shell* was performed using CaCl_2_, MgCl_2_, and ZnCl_2_

### 2.5. Chemical Activation of Chrysophyllum albidum Shells Using CaCl_*2*_, MgCl,_*2*_ and ZnCl_*2*_

The chemical activation of the pyrolised and powdered* Chrysophyllum albidum* shell was performed using 0.5M aqueous solutions of each CaCl_2_, MgCl_2_, and ZnCl_2_. 20g of the pyrolised powdered shells was weighed and added to 250ml of the various activation chemical aqueous solutions. The mixtures were then agitated with a hot plate agitator at 200rpm and 60°C for four hours. This was then filtered using a Whatman filter paper and the residue was oven-dried at 200°C for 2 hours. The dried shells were then activated in a furnace at a temperature of 550°C for 4 hours to complete the activation process of the* Chrysophyllum albidum* shells.

### 2.6. Adsorption Tests

Adsorption tests were carried out in 2L Erlenmeyer flask using 1g each of shells activated with CaCl_2_, MgCl_2_, and ZnCl_2_. Methyl orange with a concentration of 0.2mg/L and 0.5mg/L was prepared and used as the adsorbent for this study. 1g of the activated shell was weighed and dissolved in a litre of the solution and stirred using a laboratory stirrer at 250rpm. 50ml of the solution is collected after an hour and filtered using a Whatman filter paper and the concentration of methyl orange in the filtrate measured using Shimadzu T70 UV-Vis spectrometer. The data obtained were fitted to adsorption isotherm models. The initial concentrations of the methyl orange are 0.2mg/l for CaCl_2_ and MgCl_2_, respectively, and 0.5mg/l for ZnCl_2_.

## 3. Calculations

The percentage of removal of sorbent was calculated using

(1) $$\begin{array}{c} R\%=\frac{C_{o}-C_{t}}{C_{o}}\times 100, \end{array}$$

where

  R% is the percentage recovery of methyl orange from the solution
  C_O_ is the initial concentration of methyl orange in solution
  C_t_ is the concentration of methyl orange at time t.

## 4. Sorption Kinetics

### 4.1. Pseudo-First-Order Kinetic Model

It is represented by the following:

(2) $$\begin{array}{c} \frac{{dq}_{t}}{dt}=k_{1}\left(\;q_{e}-q_{t}\;\right). \end{array}$$

Integrating the above equation with boundary conditions of* t=0, q*_*t*_*=0,* and* t=t, q*_*t*_*=q*_*t*_ gives the following:

(3) $$\begin{array}{c} \log\left(\;q_{e}-q_{t}\;\right)=\log q_{e}-\left(\;\frac{k_{1}}{2.303}\;\right)t, \end{array}$$

where* q*_*e*_ and* q*_*t*_ are the amounts of dye adsorbed at equilibrium and at time* t *(mg/g), respectively,* t* is the contact time (min), and* K*_*1*_ is the pseudo-first-order rate constant (/min). The straight-line plot of log (*q*_*e*_*-q*_*t*_) against* t* gives log (*q*_*e*_) as slope and intercept equal to* k*_*1*_*/2.303*. Hence, the amount of solute sorbed per gram of sorbent at equilibrium (*q*_*e*_) and the first-order rate constant (*k*_*1*_) can be evaluated from the slope and intercept.

### 4.2. Pseudo-Second-Order Kinetic Model

This is represented by the following:

(4) $$\begin{array}{c} \frac{{dq}_{t}}{dt}=k_{2}{\left(\;q_{e}-q_{t}\;\right)}^{2}. \end{array}$$

Integrating the above equation with boundary conditions of* t=0, q*_*t*_*=0,* and* t=t, q*_*t*_*=q*_*t*_ gives the following:

(5) $$\begin{array}{c} \left(\;\frac{t}{q_{t}}\;\right)=\frac{1}{k_{2}q_{e}^{2}}+\frac{1}{q_{e}}t, \end{array}$$

where* k*_*2*_ represents the rate constant and* q*_*t*_ is the uptake capacity at any time (t).

## 5. Results and Discussion

### 5.1. Oil Characterisation

Physical and chemical parameters of the African Star Apple seed oil (*Chrysophyllum albidum*) are presented in Table 1.

The oil extracted from the African Star Apple seeds (*Chrysophyllum albidum*) with hexane using the soxhlet apparatus was physically and chemically analyzed and gave the following results as presented in Table 1.

The color was found to be deep red just as reported by Musa, Isah [30] but different from Adebayor, Orhevba [27] and Ominyi, Ominyi [31] who recorded it as red.

#### 5.1.1. Oil Yield

The oil yield was calculated based on the differences in weight of the sample and thimble before and after extraction:

(6) $$\begin{array}{c} Y\%=\left(\;\frac{Wi-Wf}{Wi}\;\right)\times 100, \end{array}$$

where

  W_*i*_ is the weight of thimble and sample before extraction
  W_*f*_ is the weight of thimble and sample after extraction.

The oil yield was very low with a value of 11.6% as compared to that of 12% recorded by Adebayor, Orhevba [27], 8.05% and 12.70% for [30]. This indicates that the seed may not be a good source of abundant oil. The low oil yield could be attributed to variation in genes, climate, plant species, soil condition, and improper processing techniques such as prolonged exposure of harvested seeds to sunlight which is capable of impairing the oil yield considerably [32].

#### 5.1.2. Refractive Index

The refractive index indicates the level of optical clarity of the crude oil sample relative to water. The refractive index of the extracted oil was 1.464 which agreed with that of 1. 46 and 1.672 at 31.2°C for Adebayor, Orhevba [27]. It is also not as thick as most drying oils whose refractive indices fall between 1.475 and 1.485 [33].

#### 5.1.3. Specific Gravity

It has a specific gravity of 0.92 at 30°C which is different from that of Adebayor, Orhevba [27] who had 0.89 at 25°C, 0.8269 at 25°C for [31]. Saponification value of oil serves as an important parameter in determining the suitability of the oil for soap making [34].

#### 5.1.4. Acid Value

The acid value is an important indicator of oxidation of the oil. It is the weight (mg) of potassium hydroxide required to neutralize the free acid in 1 g of the oil. In good oil, the acid value should be very low (< 0.1) and an increase in acid value is an indicator of oxidation of the oil which may lead to gum and sludge formation beside corrosion. The acid value was also found to be 7.72 mg/KOH/g. This is different from values of 2.57 mg/KOH/g by Musa, Isah [30], 4.50 mg/KOH/g for Adebayor, Orhevba [27], and 19.70 mg/KOH/g for Ominyi, Ominyi [31].

#### 5.1.5. Saponification Value

Saponification value (SV) is related to the average molecular mass of fatty acid in the oil sample. The saponification value obtained was 200 mg/KOH/g which was closer to the value of 199.50 mg/KOH/g obtained for Adebayor, Orhevba [27]. It was however higher than that of Ominyi, Ominyi [31] which was 90.71 mg/KOH/g but lower for Musa, Isah [30] who reported 228.4 mg/KOH/g. The high saponification value suggests the use of the oil in production of liquid soap, shampoos, and lather shaving creams [35, 36]. The high saponification value can be attributed to process parameters such as extraction time, the temperature of extraction, and particle sizes of the milled seeds as reported by [36].

#### 5.1.6. Free Fatty Acid

The free fatty acid of the extracted oil was 3.16 mg/KOH/g as compared to 2.25 mg/KOH/g by Adebayor, Orhevba [27] and 9.90 mg/KOH/g by [31]. Low free fatty acids content is indicative of low enzymatic hydrolysis. This could be an advantage as oil with high free fatty acids developing off-flavor during storage [36].

#### 5.1.7. Iodine Value

The iodine value is a measure of the degree of unsaturation of vegetable oils and determines the stability to oxidation and allows the overall unsaturation of the fat to be measured quantitatively [37]. The iodine value of the extracted oil was measured and found to be 72.8 mg/KOH/g. This was higher than that obtained by Adebayor, Orhevba [27] and Musa, Isah [30] but lower than that obtained Ominyi, Ominyi [31].

**Figure 1:**
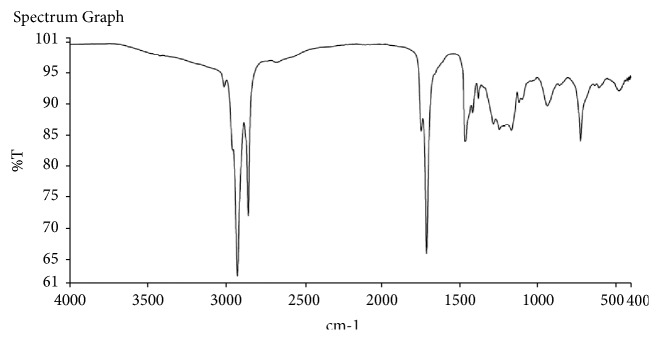
Spectral analysis of* Chrysophyllum albidum* seed oil.

FT-IR spectroscopy was used to identify the various functional groups present in the oil. A Nicolet 870 spectrometer which was equipped with a deuterated triglycine sulphate detector was used. The FTIR analysis of the extracted oil was also found to contain some functional groups. The spectral analysis as displayed in Figure 1 shows the various peaks of the functional groups present in the oil. Frequencies ranging between 3008.01cm^−1^ and 2853cm^−1^ with transmittances of 92.69 (%T) and 62.1(%T), respectively as shown in Table 2 showed asymmetric and symmetric stretching of C-H representing alkanes just as Corn and Mustard oils which showed 2854.7 – 2925.8 cm^−1^ as C-H asymmetric and symmetric stretching vibrations of the aliphatic CH2 [38]. The functional groups identified within the wavelength of 1709 – 1744 cm^−1^ were compared to oil from Chamomile and Rosemary by Anwer S. El-Badry, and Sameh S. Ali [39] as well as the Corn and Mustard oil to represent C= O ester carbonyl of triglycerides [39].

### 5.2. Adsorption Studies

**Figure 2:**
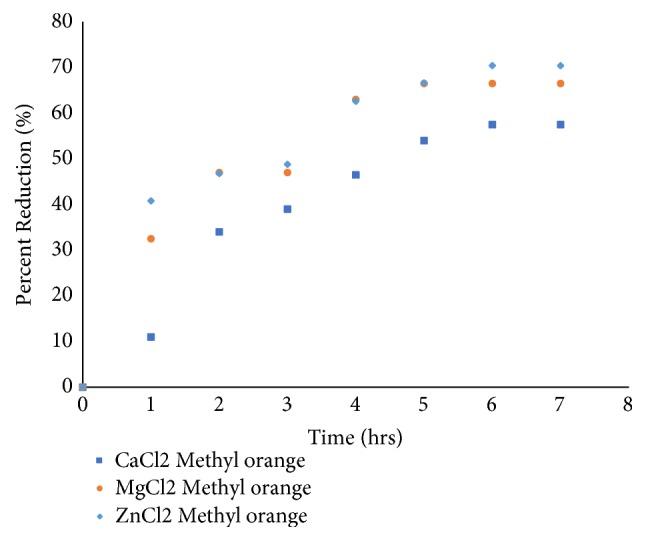
Percentage reduction of dyes with time (hrs).

The adsorption studies of Methyl orange dye solution of the fruit shell of the* Chrysophyllum albidum* activated with MgCl_2_, CaCl_2_, and ZnCl_2_ were performed.  Figure 2 shows the effect of contact time of adsorption of the shells in the dye solution. From Figure 2, it is observed that the rate of dye reduction generally increased rapidly within the first two hours of contact time for all three shells with different activation chemicals. This could be attributed to the active vacant sites within the activated shells as reported by [40]. After the first two hours, the rate of reduction of the dye decreases until equilibrium was reached after 5 hours of contact time for all the three seed shells with different activation. ZnCl_2_ and MgCl_2_ exhibit similar reduction characteristics on the dye. The overall performance of the three shells shows that ZnCl_2_ performed slightly better than MgCl_2_ in reducing the concentration of Methyl orange in the solution. Chemical activation with CaCl_2_ seems not to perform as efficient as that of the ZnCl_2_ and MgCl_2_ as shown in Figure 2.

### 5.3. Kinetic Studies

**Figure 3:**
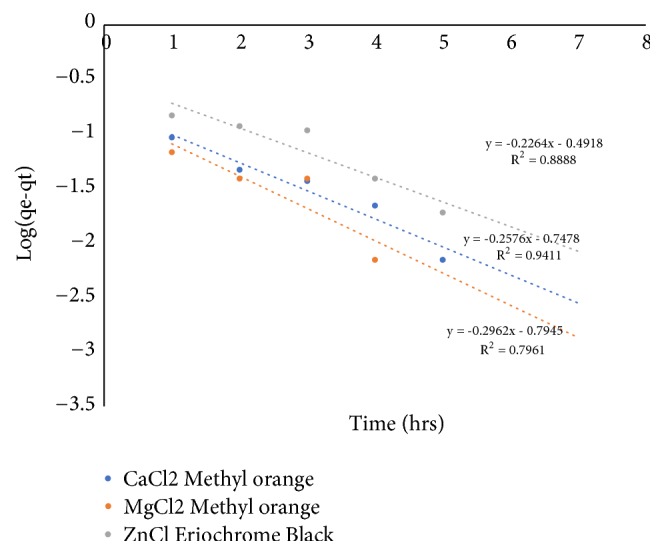
Pseudo-first-order adsorption kinetics of the dye.

**Figure 4:**
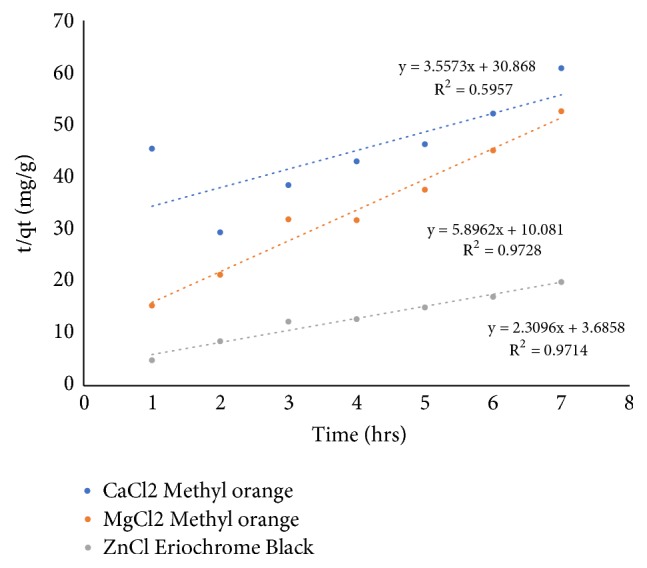
Pseudo-second-order adsorption kinetics of the dye.

Figure 3 shows the linear plot of log(q_e_ – q_t_) versus t for the Lagrangien pseudo-first-order model and Figure 4 shows the linear plot of t/q_t_ versus t for the Lagrangien pseudo-second-order model for reduction of Methyl orange using shells of* Chrysophyllum albidum* activated with CaCl_2_, MgCl_2_, and ZnCl_2_. The equilibrium rate constants and correlation coefficient for the pseudo-first-order and pseudo-second-order models are presented in Table 3. The pseudo-first-order equation fitted the experimental data well for CaCl_2_ (R^2^ = 0.94) while MgCl_2_ (R^2^ = 0.97) and ZnCl_2_ (R^2^ = 0.97) fitted the pseudo-second-order equation. On the basis of the correlation coefficient, the adsorption of Methyl orange dye from solution by shells of* Chrysophyllum albidum* activated with CaCl_2_ follows a first-order reaction pathway while the shells activated with MgCl_2_ and ZnCl_2_ follows a second-order pathway.

## 6. Conclusion

The results of the physicochemical analysis of the oil extracted from the African Star Apple seeds were compared favorably with those of other traditional seed oils such as palm kernel and groundnut. The oil yield of 11.6% was low as compared to oil from palm kernel oil (45.6%) and groundnut oil (35.76%) [41] The physicochemical properties of the African Star Apple seed oil indicated that it is nondrying (saponification value of 200mg/KOH/g) and can be used as a feedstock for production of soaps, lubricating oils, and lighting candles. However, it may not be suitable for the production of surface coatings, varnishes, and oil paints due to its nondrying attribute. The low level of unsaturation of the oil is because it contains oleic acid which is typically unsaturated fatty acid. Conclusively, the seeds may not have sufficient oil volume potential to be used as edible (domestic) and industrial oil. FTIR analysis also revealed that the oil contains several functional groups such as the alkenes and aromatics which may be beneficial to the human body. The seed shells can also be used as low-cost adsorbent when activated with CaCl_2_, ZnCl_2_, and MgCl_2_. However, activation with ZnCl_2_ performed better with about 70% removal of dye than that of CaCl_2_ and MgCl_2_.

## Figures and Tables

**Table 1:** Physical and Chemical Properties of the Extracted *Chrysophyllum albidum* seed Oil.

Property	Reported Values
Oil content^a^	11.6%
Refractive index^a^	1.464 at 30°C
Specific gravity^a^	0.92
Acid Value (mgKOH/g)^b^	7.72
Saponification Value (mgKOH/g)^b^	200.67
Free Fatty Acids (as oleic acid)^b^	3.16
Iodine Value (mg/g)^b^	72.80

Physical properties ^a^  Chemical properties ^b^

**Table 2:** Table of the FTIR results of the Oil showing the various frequencies, % transmittance and their functional assignments.

PEAKS	X (cm^−1^)	Y (%)	BOND	FUNCTIONAL GROUP
1	3008.01	92.69	C-H stretch	alkanes
2	2922.75	62.1	C-H stretch	alkanes
3	2853.48	71.81	C-H stretch	alkanes
4	1744.47	84.56	C=O stretch	carbonyls
5	1709.28	65.73	C=O stretch	Carbonyls
6	1463.81	83.85	C-H bend	Alkenes
7	1413.22	88.48	C-C stretch (in ring)	Aromatics
8	1377.63	90.8	C-H rock	alkenes
9	1281.86	86.67	C-H wag (-CH2X)	Alkyl halides
10	1242.46	85.78	C-N stretch	Aliphatic amines
11	1164.78	85.63	C-N stretch	Aliphatic amines
12	1117.12	90.16	C-N stretch	Aliphatic amines
13	936.25	89.62	O-H bend	Carboxylic acid
14	721.94	83.92	C-H rock	alkanes
15	604.69	92.5	C-Br stretch	Alkyl halides

**Table 3:** The calculated parameters of the pseudo-first-order and pseudo-second order models for the adsorption methyl orange using activated ZnCl_2_, MgCl_2_ and CaCl_2_.

Parameters	Methyl Orange	Methyl Orange	Methyl Orange
	ZnCl_2_	MgCl_2_	CaCl_2_
*Pseudo-first order kinetics*			
k_1_, min^−1^	0.52	0.68	0.59
q_t_, mg/g	0.32	0.17	0.18
R^2^	0.889	0.796	0.941

*Pseudo-second order kinetics*			
q_e_, mg/g	0.43	0.17	0.28
k_2_, g/mg min	1.45	3.44	0.41
H	0.27	0.10	0.03
R^2^	0.9714	0.973	0.596

## Data Availability

(1) The physical and chemical properties of the extracted oil data used to support the findings of this study are included within the article. (2) The Fourier Transform Infrared data used to identify the various functional groups regarding the oil of this study are included within the article. (3) The adsorption data used to support the findings of this study are included within the supplementary information file (available here).

## Supplementary Materials

Supplementary Materials This is the adsorption data that was used for this study. It shows the initial concentrations of the dye (Methyl orange) as well as the adsorbent (*Chrysophyllum albidum* seed shell) which was activated with three different chemicals (CaCl2, MgCl2, and ZnCl2). The experiment was conducted under room temperature of 25 C. The change in concentration of the dye with time could be seen in the date above.
