# Supplementary material for: Extraction and Characterisation of African Star Apple (Chrysophyllum albidum) Seed Oil and the Adsorptive Properties of the Fruit Shell in Ghana
Source: Int J Food Sci. 2019 Apr 1;2019:4959586. doi: 10.1155/2019/4959586 (PMC6463670; doi:10.1155/2019/4959586)
Supplement: Supplementary Materials — This is the adsorption data that was used for this study. It shows the initial concentrations of the dye (Methyl orange) as well as the adsorbent (Chrysophyllum albidum seed shell) which was activated with three different chemicals (CaCl2, MgCl2, and ZnCl2). The experiment was conducted under room temperature of 25 C. The change in concentration of the dye with time could be seen in the date above. [file 4959586.f1.pdf]

Supplementary Materials

This is the adsorption data was used for this study.

Initial Concentration Of dyes	0.2	0.2	0.5
Time(hours)	$CaCl_2$ Methyl orange	$MgCl_2$ Methyl orange	$ZnCl_2$ Methyl Orange
1	0.178	0.135	0.296
2	0.132	0.106	0.266
3	0.122	0.106	0.256
4	0.107	0.074	0.187
5	0.092	0.067	0.167
6	0.085	0.067	0.148
7	0.085	0.067	0.148

It shows the initial concentrations of the dye (Methyl Orange) as well as the adsorbent (*Chrysophyllum albidum* seed shell) which was activated with three different chemicals ($CaCl_2$, $MgCl_2$, $ZnCl_2$). The experiment was conducted under room temperature of 25^oC. The change in concentration of the dye with time could be seen in the date above
